# Comparative transcriptome analysis to investigate the potential role of miRNAs in milk protein/fat quality

**DOI:** 10.1038/s41598-018-24727-y

**Published:** 2018-04-19

**Authors:** Xuehui Wang, Li Zhang, Jing Jin, Anting Xia, Chunmei Wang, Yingjun Cui, Bo Qu, Qingzhang Li, Chunyan Sheng

**Affiliations:** Key Laboratory of Dairy Science, Ministry of Education, Northeast Agricultural University, Harbin, 150030 Heilongjiang China

## Abstract

miRNAs play an important role in the processes of cell differentiation, biological development, and physiology. Here we investigated the molecular mechanisms regulating milk secretion and quality in dairy cows via transcriptome analyses of mammary gland tissues from dairy cows during the high-protein/high-fat, low-protein/low-fat or dry periods. To characterize the important roles of miRNAs and mRNAs in milk quality and to elucidate their regulatory networks in relation to milk secretion and quality, an integrated analysis was performed. A total of 25 core miRNAs were found to be differentially expressed (DE) during lactation compared to non-lactation, and these miRNAs were involved in epithelial cell terminal differentiation and mammary gland development. In addition, comprehensive analysis of mRNA and miRNA expression between high-protein/high-fat group and low-protein/low-fat groups indicated that, 38 miRNAs and 944 mRNAs were differentially expressed between them. Furthermore, 38 DE miRNAs putatively negatively regulated 253 DE mRNAs. The putative genes (253 DE mRNAs) were enriched in lipid biosynthetic process and amino acid transmembrane transporter activity. Moreover, putative DE genes were significantly enriched in fatty acid (FA) metabolism, biosynthesis of amino acids, synthesis and degradation of ketone bodies and biosynthesis of unsaturated FAs. Our results suggest that DE miRNAs might play roles as regulators of milk quality and milk secretion during mammary gland differentiation.

## Introduction

All female mammary breast tissue secretes milk as liquid food to support offspring growth and development and to provide necessary nutrients and important active substances, such as immune factors, growth factors, and enzymes. Therefore, given human nutritional requirements and the need for animal welfare, determining how to maximize milk yield and improve milk quality without affecting the health of the lactating animals, including their reproductive capacity and lifespan, has been the mission of scientific researchers.

MicroRNAs (miRNAs) are small molecules encoded in the genomes of higher eukaryotes that function similar to siRNAs. miRNAs interact with their target gene mRNAs through the RNA-induced silencing complex, which degrades mRNAs or hinders mRNA translation^1,2^. High-throughput sequencing provides a unique opportunity to catalog the RNA expression profile and study miRNA-mRNA interactions in mammary tissue. Li *et*. *al*. performed miRNA sequencing and showed that miRNAs are related to lactation^3^. In addition, miRNA-seq of goat mammary gland tissue indicated that miRNAs are involved in the lactation process^4^. The first miRNA-seq profiling study of liver tissue revealed that miR-143 is involved in lipid metabolism and negative energy balance in the postpartum dairy cow liver^5^. The breast is a dynamic organ that experiences significant developmental changes during pregnancy, lactation, and the post-lactation processes. Transcriptome sequencing can be used to analyze gene expression in the mammary gland and to identify the major genes associated with lactation. Genes differentially expressed (DE) in the mammary gland between lactating and non-lactating cows are primarily involved in regulating the expression of protein and fat synthesis during lactation, and these genes include the kinase mechanistic targets of rapamycin complex 1 (*mTORC1*), lipoprotein lipase (*LPL*), stearoyl-coenzyme A desaturase (*SCD*) and long-chain fatty acid (FA) CoA synthetase (*ACSL*). Wang *et* *al*. reported that low-quality forage diets influenced the expression of the feed and nitrogen efficiency-associated miRNAs miR-21-3p and miR-2285f, which regulated amino acid transport by targeting *ATP1A2* and *SLC7A8*, thereby influencing milk protein metabolism and quality^6^.

In this study, to investigate the potential role of miRNAs in milk quality, we constructed interaction networks between miRNAs and mRNAs and performed a comprehensive analysis consisting of RNA-binding protein immunoprecipitation (RIP) sequencing (RIP-seq), miRNA-seq, and mRNA-seq. Functional enrichment analyses revealed several novel miRNAs involved in epithelial cell development, mammary gland development, regulation of cell proliferation, amino acid transport, and lipid metabolism. These findings implied that these novel miRNAs may play important roles in lactation. Therefore, this evidence provides new hypotheses on lactation for further exploration.

## Results

### miRNAome profiling

**Figure 1:**
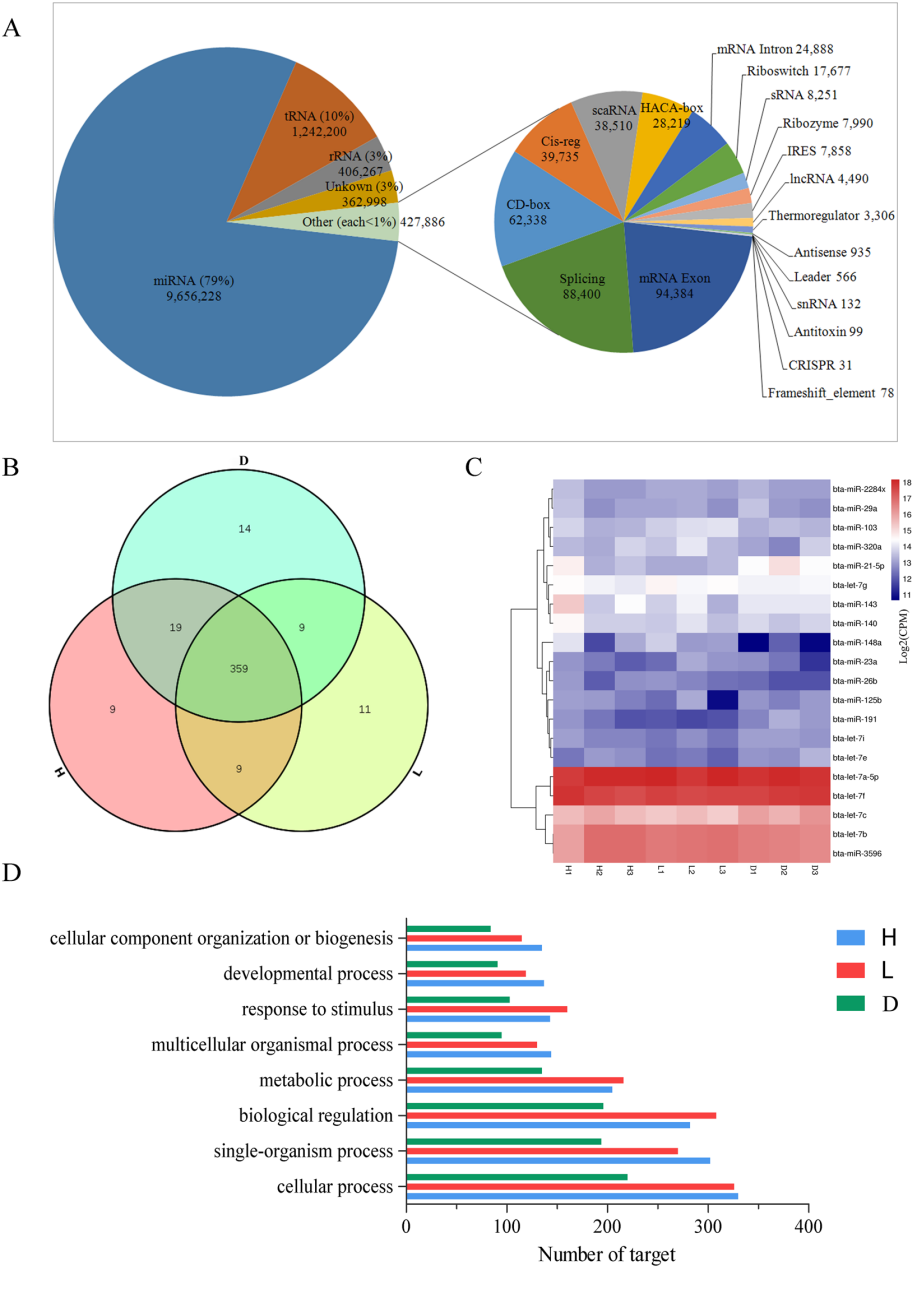
(**A**) The results of miRNA sequencing analysis revealing various small RNA species. (**B**) Commonly expressed and period-specific known miRNAs identified in the three groups. (**C**) Expression of the top 20 miRNAs commonly expressed in the three groups. (**D**) GO annotation analysis of targets of period-specific miRNAs in the three groups.

The miRNA profiles were generated by sequencing nine small RNA libraries prepared from high-protein/high-fat mammary gland tissues, low-protein/low-fat mammary gland tissues and dry period mammary gland tissues. In total, 88.3 million out of 119.7 million clean reads (13 million reads per tissue) were mapped to small RNA sequences in Btau_4.6.1. Mapping statistics showed an average mapped rate of 91.9% with a standard deviation of 2.6% (Table 1). The remaining reads that were unmapped to known miRNAs were used to predict novel miRNAs using miRDeep2^7^. In order to predict novel miRNAs with high confidence, only those with a miRDeep2 score higher than five and present in at least three libraries with at least 10 total counts were retained as novel miRNAs^8^. Additionally, the clean reads were annotated to ncRNA families using Rfam^9^. The clean reads were classified into different small RNA categories; an average of 79% clean reads was aligned to miRNA sequences, 10% to tRNA sequences, and 3% to rRNA sequences, respectively. The remaining clean reads were aligned to non-coding RNAs (sRNA, snRNA, scaRNA) and others (Fig. 1A). A total of 430 known miRNAs (3p or 5p) were identified which were expressed at an abundance of more than one count per million (CPM) in at least three libraries (known miRNA profile listed in Table S3a). Based on the above test, 29 novel miRNA precursors coding for 28 mature miRNAs were identified in this study. Novel miRNAs that did not meet our criteria were retained as candidate miRNAs for further (reference) study. Of these, several were more abundant in the non-lactation group (D group) than in the lactation groups (H and L groups), while one, chr_9_17896_star, was more abundant in the dry period than in the lactation period (log_2_ fold change ≥ 1; FDR ≤ 0.05). Several miRNAs, such as chr_19_34912_mature, were more abundant in the lactation group (log_2_ FC ≥ 1; FDR ≤ 0.05). This differential enrichment of candidate novel miRNAs indicated that these miRNAs might regulate mammary gland development and milk quality (novel miRNA profile listed in Table S3b, miRDeep2 details shown in S3c, S3d, S3e).

**Table 1:** Number of clean reads generated from each group and mapping statistics.

Group	Total clean reads	Reads mapped	Mapped rate (%)
H	35,658,592	33,193,931	93.3%
L	42,930,552	37,813,438	88.3%
D	41,111,160	37,852,851	91.8%

### Groups of commonly and uniquely expressed miRNAs

In this study, a total of 359 miRNAs were commonly expressed in all three groups (Fig. 1B). Among the top 20 most highly expressed miRNAs in the three groups, bta-let-7a-5p and bta-let-7f were highly expressed in all groups (Fig. 1C). The predicted function of the top 20 commonly expressed miRNAs was related to cellular process, biological regulation, single organism process, and metabolic process (Fig. 1D).

miRNAs uniquely expressed in one specific period were defined as period-specific miRNAs. In total, 9, 11 and 14 period-specific miRNAs were identified in the H group, the L group, and the D group, respectively. Functional analysis showed that these period-specific miRNAs was related to cellular process, single-organism process, biological regulation, and metabolic process (Fig. 1D). The differential expression of period-specific miRNAs among groups was subsequently examined.

### Genome-wide identification of DE miRNAs and mRNAs

**Figure 2:**
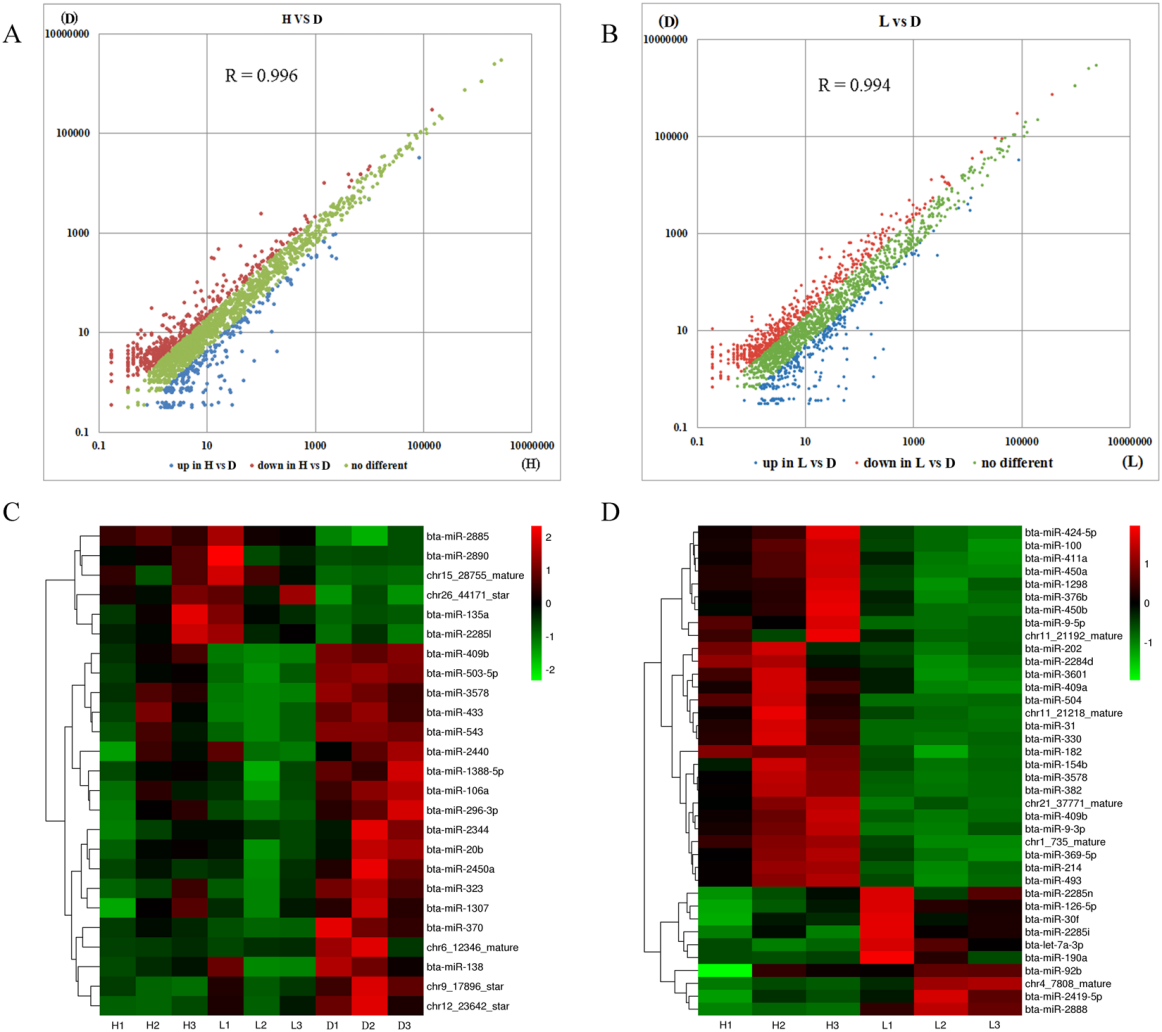
(**A**) The correlations between libraries H and D. (**B**) The correlations between libraries L and D. (**C**) The 25 core DE miRNAs between lactation and non-lactation. (**D**) miRNAs DE in the high-protein/fat group compared to the low-protein/fat group.

The correlations between libraries (H and D groups) were calculated using the normalized counts of expressed miRNAs. The Pearson correlation between these two libraries was R = 0.996, indicating a strong correlation (Fig. 2A). The expression of 41 miRNAs was significantly different (FDR ≤ 0.05) between the H and D groups (Table S3f). Of these, 14 were up-regulated and 27 were down-regulated in the H group compared with the D group. All of the DE miRNAs exhibited fold changes ≥ 2 or ≤ −2 with a FDR ≤ 0.05. The correlation between the L and D libraries was also higher: R = 0.994 (Fig. 2B). We identified 137 DE miRNAs, including 101 down-regulated and 36 up-regulated miRNAs, in the L group compared to the D group (Table S3f). A core of 25 shared DE miRNAs was identified in both lactation groups (H and L groups; Fig. 2C). Of these, 6 miRNAs were more abundant during the lactation period than during the dry period, and 19 miRNAs were more abundant in the dry period.

The Pearson correlation between the H and L libraries was also calculated using the same method. The result of R = 0.998 indicated a strong correlation between the two libraries. To identify miRNAs that were more abundant in either the H group or the L group, we performed differential expression analysis on each miRNA by comparing the expression levels between the two groups and identified characteristic candidate novel and known miRNAs associated with milk quality. We found that 28 miRNAs exhibited increased abundance in the H group and that 10 miRNAs showed increased abundance in the L group (FDR ≤ 0.05; log_2_ fold change ≥ 1; Fig. 2D; Table S3f). Moreover, 944 mRNAs showed differential expression between the H and L groups: 455 differentially expressed genes (DEGs) were more abundant in the L group, and 489 DEGs were more abundant in the H group (Table S3g). In addition, expressed mRNA data is listed in table S3h.

### Prediction of targets and functional annotation of DE miRNAs between lactation and non-lactation

**Figure 3:**
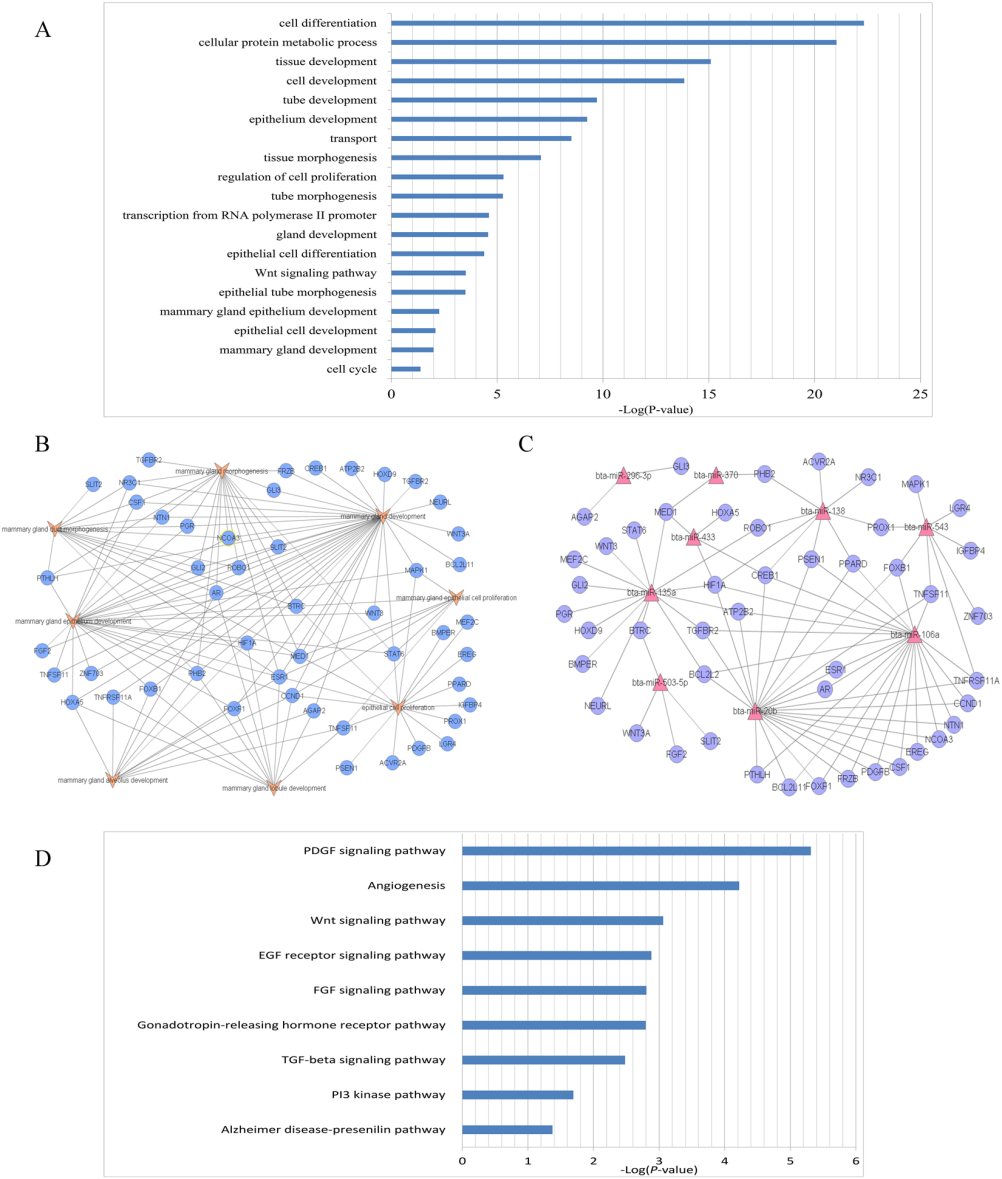
Functional annotations and enriched canonical pathways of the 25 targeted core DE miRNAs. (**A**) Functional annotation of all the targets by 25 core DE miRNAs. (**B**) A relationship network showing the predicted genes corresponding to the 25 core DE miRNAs between lactation and non-lactation, which are highly related to mammary gland development and mammary epithelial cell proliferation. (**C**) These relationships of these target genes with DE miRNAs. (**D**) Enriched canonical pathways related to lactation initiation.

We identified 25 miRNAs, including 6 up-regulated and 19 down-regulated, with significantly different expression in the lactation groups compared with the non-lactation group. These 25 core DE miRNAs identified in this study tended to have key roles in regulation of mammary development and lactation. Targets of these core miRNAs were predicted using TargetScan Release 7.1 software^10^. The 25 core miRNAs were predicted to target 3416 genes with high confidence. Next, we performed functional significance analysis of the targets using PANTHER^11^. The functions most significantly enriched in target genes of the core DE miRNAs were epithelial cell development, mammary gland development and regulation of cell proliferation (Fig. 3A). We selected a set of predicted target genes related to mammary gland development and mammary epithelial cell proliferation to generate a relationship map (Fig. 3B) and a miRNA-mRNA network (Fig. 3C). The results indicated that bta-miR-106a, bta-miR-20b and bta-miR-135a may play roles as regulators of mammary gland development and mammary epithelial cell proliferation. Furthermore, pathway enrichment analysis shown that the EGF receptor, FGF signaling, and TGF-beta signaling pathways were enriched, and these enriched pathways associated with mammary development and lactation (Fig. 3D).

### Prediction of targets and functional annotation of DE miRNAs

In order to explore the possible roles of the 38 DE miRNAs in milk quality, we used PANTHER software to analyze gene targets of DE miRNAs and to investigate the functions enriched in these miRNAs that might impact milk quality. Our analysis indicated that the 38 DE miRNAs targeting 8119 genes. The functions most significantly enriched in target genes of DE miRNAs were cell differentiation, cellular development process, regulation of gene expression, and regulation of cellular biosynthetic process, followed by gene expression (Figure S1). Further, we explored the pathways associated with lipid metabolism, carbohydrate metabolism, and amino acid metabolism (Figure S1).

### Enrichment analysis of differentially expressed genes

**Figure 4:**
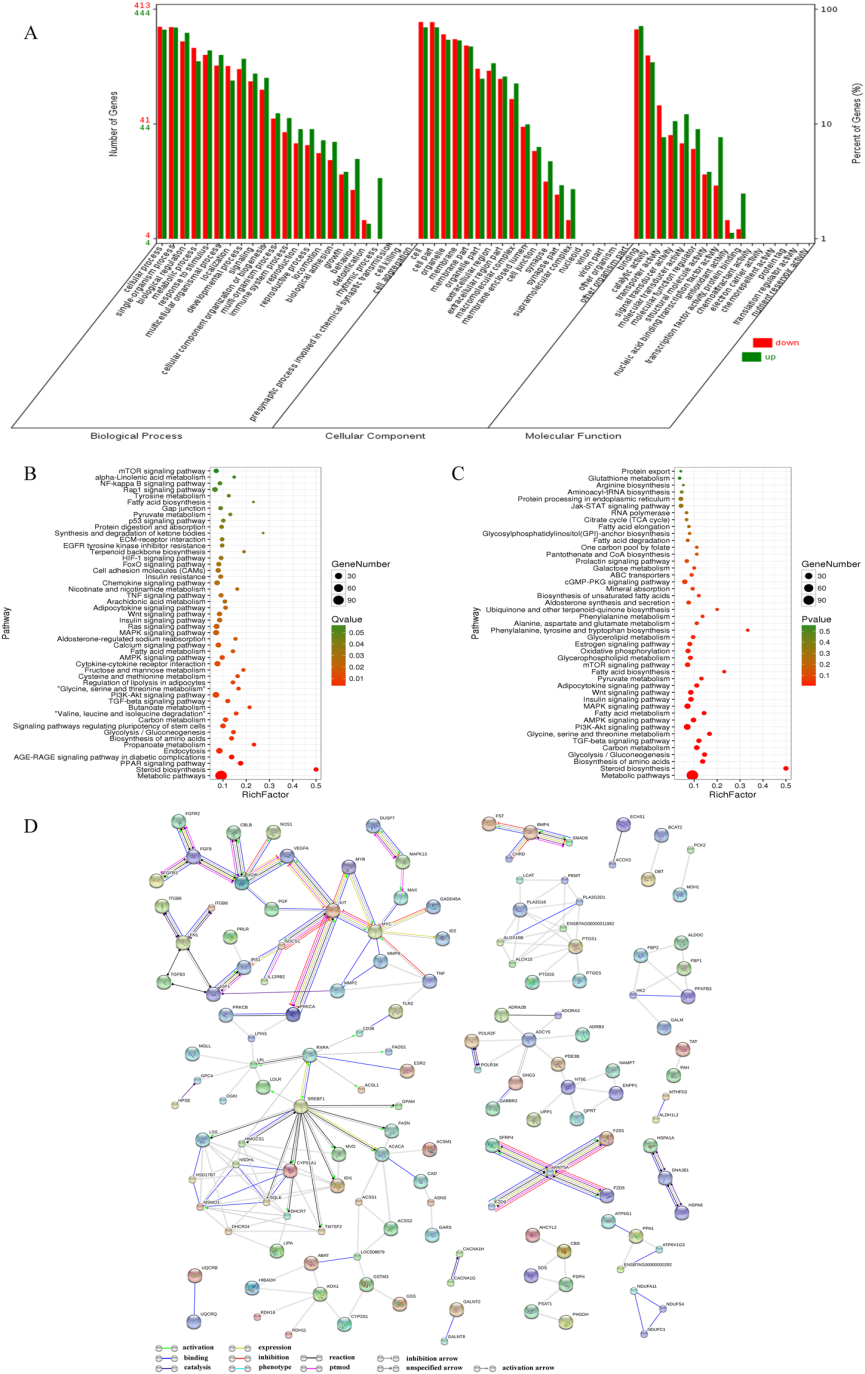
(**A**) GO classification of the DEGs detected in the H and L groups. The green column represents genes up-regulated in the H compared to the L group, and the red column represents genes down-regulated in the H group compared to the L group. (**B**) Top annotated KEGG pathways sorted by *P* value for DEGs in the H relative to the L. The circle size represents the number of genes for each pathway, and the circle color represents the degree of pathway enrichment. (**C**) Pathways related to protein and fat metabolism were selected. The circle size represents the number of genes for each pathway, and the circle color represents the degree of pathway enrichment. (**D**) Gene-Act network of DEGs between the H and L groups according to the pathways in the STRING database.

A Gene Ontology (GO) enrichment analysis of DEGs was performed to identify significantly enriched GO terms. GO terms were assigned to 455 down-regulated and 489 up-regulated DEGs (Fig. 4A). We found that most DEGs were classified into the categories of catalytic activity, transporter activity, signal transduction activity, transcription factor activity, and protein binding. Pathway enrichment analysis indicated that FA metabolism and biosynthesis of amino acids were significantly enriched in these DEGs; the top significantly enriched KEGG pathways were sorted by *P* value (Fig. 4B). In addition, the pathways related to protein and fatty metabolism, including the prolactin signaling pathway and most signal transduction pathways, were examined (Fig. 4C). Furthermore, protein-protein interaction analysis was performed using the STRING database, and a Gene-Act network was constructed to explore the relationship among DEGs. The results indicated that sterol regulatory element binding transcription factor 1 (*SREBF1*), kinase insert domain receptor (*KDR*), KIT proto-oncogene receptor tyrosine kinase (*KIT*), insulin like growth factor 1 (*IGF1*), v-myc avian myelocytomatosis viral oncogene homolog (*MYC*), adenylate cyclase 5 (*ADCY5*), and acetyl-CoA carboxylase alpha (*ACACA*) were at the core of the interaction network in the comparison between the H and L groups (Fig. 4D).

### Integrative analysis of DE miRNAs and DE mRNAs

**Figure 5:**
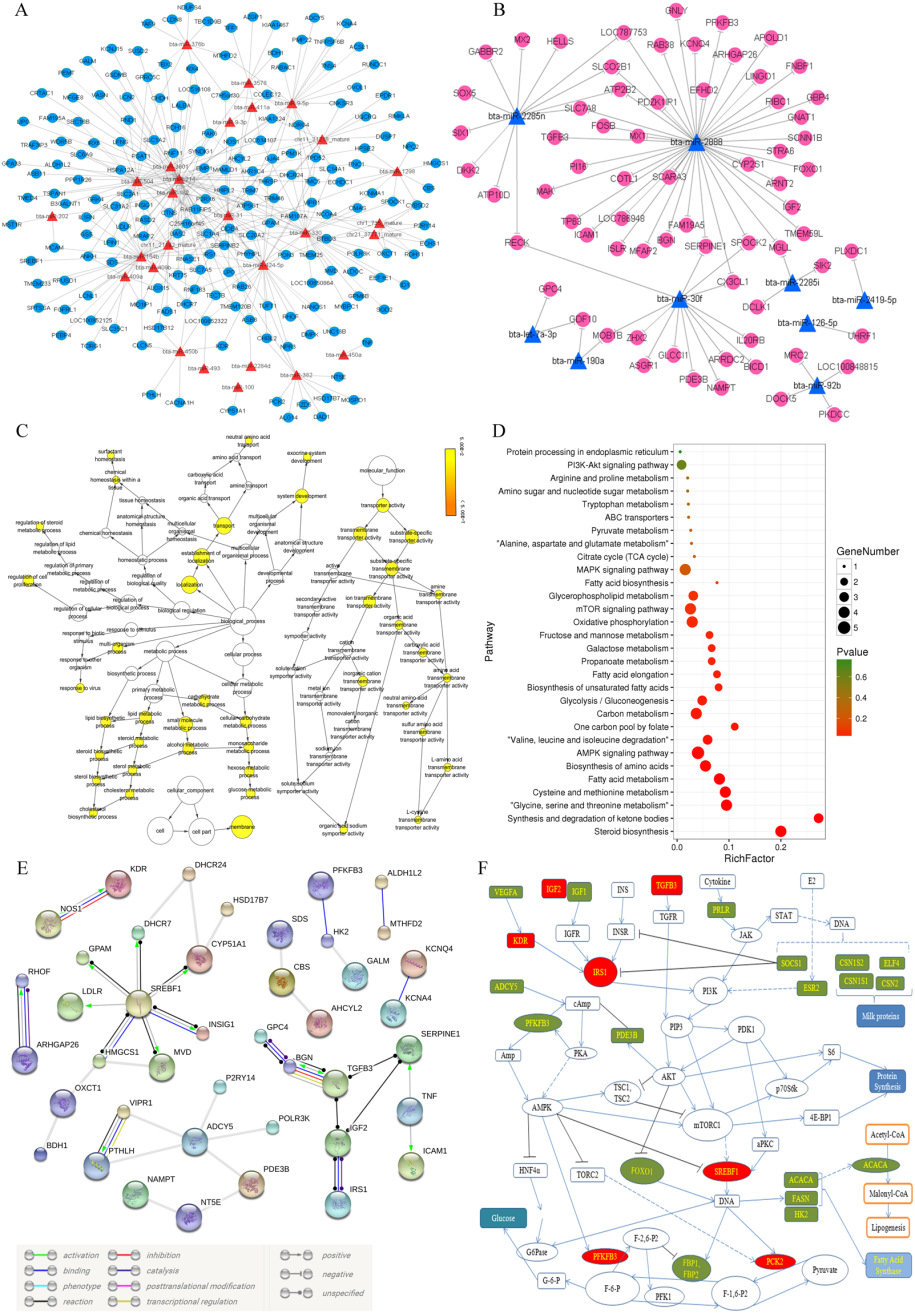
Co-expression networks of DE miRNAs and mRNAs from the H group in comparison with the L group. (**A**) Red triangles represent up-regulated miRNAs, and blue circles represent down-regulated mRNAs. (**B**) Blue triangles represent down-regulated miRNAs, and red circles represent up-regulated mRNAs. (**C**) GO enrichment analysis of DE mRNAs in miRNA-mRNA pairs. The circle size represents the number of genes for each GO term, and the circle color represents the degree of GO term enrichment. (**D**) KEGG pathway enrichment analysis of DE mRNAs in miRNA-mRNA pairs. The circle size represents the number of genes for each pathway, and the circle color represents the degree of pathway enrichment. (**E**) Gene-Act network of DE mRNAs in miRNA-mRNA pairs according to the pathways in the STRING database. (**F**) Some of the biological pathways involved in milk protein and fat metabolism. The red and green boxes indicate genes DE in the H compared to the L group. The red boxes represent DEGs that may be targets of DE miRNAs.

Translation and mRNA stability are regulated by miRNAs. Therefore, miRNAs may affect the quality of milk by regulating genes associated with milk quality. Based on this possibility, we assessed the relationship between differential miRNA expression and mRNA enrichment. Using the miRNA and mRNA profiles from the H group and the L group, we identified putative negative regulatory miRNA-mRNA interactions. We found inverse associations between the abundances of 38 DE miRNAs and 253 DE mRNAs. Networks of miRNA-mRNA pairs were constructed to better understand the relationships between these novel miRNAs and mRNAs (Fig. 5A,B).

A GO enrichment analysis of the 253 putative genes was performed to identify GO terms with high confidence. We found that lipid biosynthetic process and amino acid transmembrane transporter activity were the most enriched GO terms (Fig. 5C). Pathway enrichment analysis indicated that FA metabolism, biosynthesis of amino acids, synthesis and degradation of ketone bodies and biosynthesis of unsaturated FAs were significantly enriched in the putative genes (Fig. 5D). Additionally, protein-protein interaction analysis indicated that *SREBF1* was at the core of the interaction network of DE miRNAs and DE mRNAs between the H and L groups (Fig. 5E). Gene pathway network analysis indicated that signal transduction, cell cycle and metabolism were the most important pathways related to putative DE genes between the H and L groups (Figure S2). Gene correlation analysis based on the KEGG pathway database showed that most genes share an association with the same pathway in the same database (Figure S2). Further, we list a set of the important putative genes in miRNA-mRNA pairs that are involved in insulin receptor signaling pathway, lipid metabolic process, transporter activity, AMPK signaling pathway, mTOR signaling pathway, MAPK signaling pathway, and PI3K-Akt signaling pathway (Table 2).

**Table 2:** List of some of the important DEGs negatively regulated by DE miRNAs.

Term	Gene symbol	*P* value
insulin receptor signaling pathway	*IGF2, SIK2, FOXO1, IRS1, SREBF1*	3.33E-02
lipid metabolic process	*ABCD1, ACSL1, ALOX15, CACNA1H, CMAS, CYP51A1, DHCR24, DHCR7, ECHS1, FADS1, GPAM, HMGCS1, HSD17B12, IRS1, HSD17B7, IL1RN, INSIG1, LDLR, MGLL, MVD, NPC2, PEMT, RDH11, SLC35C1, SPTSSA, SREBF1*	2.13E-04
transporter activity	*ATP2B2, ABCD1, ANKH, ATP10D, ATP5G1, CACNA1H, CLCN5, CTNS, KCNA4, KCNJ15, KCNMA1, KCNQ4, LCN2, LCNL1, P2RX6, SCNN1B, SLC14A1, SLC1A2, SLC1A4, SLC20A2, SLC2A1, SLC31A2, SLC6A9, SLC7A8, SLCO2B1, STRA6, UQCRQ*	4.59E-03
AMPK signaling pathway	*IRS1, SREBF1, FOXO1, PFKFB3, PCK2*	6.88E-03
mTOR signaling pathway	*FZD5, IRS1, SLC7A5, TNF*	5.94E-02
MAPK signaling pathway	*DUSP7, CACNA1H, TNF, TGFB3*	2.16E-01
PI3K-Akt signaling pathway	*IRS1, KDR, PCK2*	6.22E-01

Based on the above analysis, we found that some of the DEGs, including target genes of DE miRNAs, were involved in various classic signaling pathways involved in the synthesis of lipids and proteins. To more comprehensively study the miRNA-mediated regulation of milk protein and milk fat, a simple regulatory relationship network was constructed (Fig. 5F). The results indicated that *PCK2, PFKFB3, SREBF1, IRS1, KDR, TGFB3* and *IGF2* are involved in this regulation and can be regulated by miRNAs.

### Validation of miRNAs targets by RIP

To further validate the candidate miRNA-mRNA interactions in mammary gland tissue, we performed co-immunoprecipitation with high-throughput sequencing-mediated profiling of mRNA expression to identify relevant miRNA targets. The global “miRNAome” and “targetome” of tissues were determined to better understand the functional roles of miRNAs. Immunoprecipitation combined with fragment analysis revealed 104 unique annotated protein-coding genes. Moreover, 15 of these genes showed significantly different expression between the H and L groups (Table S2). These genes, including *LALBA, RHOF, MFGE8, TMEM120B* and *GPAM*, matched the above-described negative regulatory relationship miRNAs.

### Validation of RNA sequencing data by quantitative real-time PCR (qPCR)

qPCR was used to validate the RNA sequencing data in this study. The miRNAs of chr9_17896_star, chr19_34912_mature, bta-miR-200b, bta-miR-199a-3p, bta-miR-29c, bta-miR-129-3p, bta-miR-34a, bta-miR-92b, bta-miR-126-5p, bta-miR-2888, bta-miR-214, bta-miR-424-5p, bta-miR-382 and bta-miR-330 were examined, and the mRNAs of *LALBA*, *RHOF*, *MFGE8*, *TMEM120B* and *GPAM* were examined. The trends in the expression of the selected miRNAs and mRNAs were generally similar to the results from miRNA-seq, mRNA-seq or RIP-seq. Thus, these results confirm that the sequencing results are reliable (Figure S3).

## Discussion

To date, miRNAs have been extensively studied in animals^12,13^. Specifically, the functions of miRNAs in animal development and human diseases have been widely studied^14^. miRNAs play an important role in the functioning and performance of livestock production, including the regulation of muscle development and hypertrophy, adipose tissue growth, oocyte maturation and early embryonic development in cattle, chickens, pigs, and sheep^15^. It has been reported that miRNAs play roles in lactation initiation^3^ and that milk protein and fat synthesis are affected by the diet^6^.

In this study, we focused on miRNAs in the mammary gland from three groups (including a dry period group) of dairy cows and their roles in milk protein/fat. The miRNAome was examined using a high-throughput small RNA sequencing method. Generally, a RNA sequencing tag density of 1–2 million reads is sufficient for miRNA expression profiling, and a tag density of 2–5 million reads is sufficient for discovery applications^16^. We generated an average of more than 12 million reads for each library in this study. In addition, we conducted integrative miRNA-mRNA expression analysis to explore the potential impacts of miRNAs on milk protein/fat. These data provide genome-wide insight into miRNA expression and its alteration of milk protein/fat from the bovine mammary gland.

We identified 430 known miRNAs with an average abundance of greater than one CPM in at least three libraries; this set of miRNAs accounted for 54.05% of all known bovine miRNAs deposited in miRBase21^17^. A total of 359 miRNAs were found to be expressed at different lactation stages in the mammary gland. These miRNAs might regulate mammary gland development and lactation processes, thereby affecting milk quality. Further functional analysis of period-specific miRNAs and DE miRNAs between groups that cellular process and metabolic process were most enriched in these miRNAs. Therefore, the organization of cellular function in different periods could be regulated by distinct miRNAs. This characteristic may contribute to the different physiological functions observed between periods. The highly expressed miRNAs observed in our study (Fig. 1C) were detected in previous reports in the lactating mammary gland^3,18,19^, bovine ovary^20^ and dairy cow liver^5^. Additionally, our results indicated that *PCK2, PFKFB3, SREBF1, IRS1, KDR, TGFB3* and *IGF2* are prominently involved in regulating FA synthase and milk protein synthesis (Fig. 5F), which are target genes of the identified DE miRNAs (Fig. 5A,B). Combined with previously reports, our study suggests that these highly expressed miRNAs may be involved in metabolic, developmental and biological regulation, ultimately affecting milk synthesis and secretion. There were 41 DE miRNAs in the H group compared to the D group and 137 DE miRNAs in the L group compared to the D group. Only a few DE miRNAs overlapped between our current study and previous studies^3,6,19^. Despite this discrepancy, it is reasonable to hypothesize that there are individual differences between animals and differences in the processing methods and model algorithms used^21^. However, in our study, several previously reported miRNAs^19,22–24^, such as bta-miR-100, miR-1388-3p, miR-141, miR-148a, miR-181c, miR-181d, miR-199a-3p, miR-199b, miR-200b, miR-221, miR-32, miR-362-3p, miR-375, miR-20b, miR-215 and miR-9-5p, were also detected.

An interesting result is that 25 miRNAs were differentially expressed between the H and D groups and between the L and D groups. These results suggest that these miRNAs are likely involved in the regulation of lactation initiation in the mammary gland. Autocrine production of prolactin by mammary epithelial cells is required for their terminal differentiation during late pregnancy as well as the initiation of lactation at parturition. Autocrine prolactin in the mammary gland is regulated by the Pten–Akt pathway. Akt1 activation is sufficient in virgin mice to up-regulate the expression of a series of genes whose expression is normally not up-regulated until the onset of secretory activation at the initiation of lactation, including *α-lactalbumin*, *ɛ-casein*, *Aldoc*, and *Elovl*^25,26^. Functional annotation of these miRNA targets revealed that functions involved in epithelial cell development, mammary gland development, regulation of cell proliferation, and gene expression were enriched in these miRNAs. Furthermore, the EGF receptor, FGF signaling, and TGF-beta signaling pathways were significantly enriched in these miRNAs (P ≤ 0.05), indicating that these miRNAs may play roles in mammary gland development and lactation initiation. Cell proliferation is critical to lactation initiation. Several of the core DE miRNAs identified in this study have previously been reported to play roles in cellular processes. There is evidence that miR-433, miR-138, mRNA-370, miR-135a and miR-296-3p are involved in cell proliferation, growth and differentiation^27–31^. Moreover, miR-103^32^, miR-2885, miR-135a and miRNA-370 are involved in metabolism of glucose and lipids^33–35^. Furthermore, miR-433 affected cell differentiation by regulating the transcript level of Runx2^36^. miR-138 might promote proliferation and migration by regulating the expression of sirtuin 1 (*SIRT1*)^37^. miR-370 may play important roles in the morphogenesis of diverse organs by modulating the expression of DNA (cytosine-5)-methyltransferase 3 alpha (*DNMT3A*)^38^, which affects the lactation activity of dairy cow mammary epithelial cells (DCMECs). miR-29s participates in the regulation of milk synthesis and secretion by directly targeting DNMT3A and DNA (cytosine-5)-methyltransferase 3 beta (*DNMT3B*) to reduce DNA methylation^39^. miR-135a attenuated the insulin-stimulated phosphorylation and activation of phosphatidylinositol 3-kinase p85α (*PI3Kp85α*) and v-Akt murine thymoma viral oncogene homolog (*AKT*) as well as glucose uptake, and the level of its target insulin receptor substrate 2 (*IRS2*) regulates insulin signaling^40^. Insulin and insulin-like growth factor (*IGF*) have been reported to function in the regulation of milk production^41^. For example, insulin affects glucose utilization and glucose production during early lactation and may also participate in a mechanism of storing gluconeogenic substrates during early lactation^42^. Insulin increases the utilization of acetate for lipid synthesis while decreasing lipolysis in adipose tissue^43^; additionally, insulin regulates milk protein synthesis in dairy cows^44^. Moreover, in this study, several important lactation initiation factors, including EGF receptor (*EGFR*), *FGF1*, growth hormone receptor (*GHR*), *IGF1*, prolactin receptor (*PRLR*), signal transducer and activator of transcription 3 (STAT3), transforming growth factor-beta receptor 1 (*TGFBR1*) and wingless-type MMTV integration site family member 3 (*WNT3*) were predicted to be targets of DE miRNAs. However, although there is evidence that the core DE miRNAs may play a role in lactation initiation, their mechanisms require further verification.

The DE miRNAs were more likely to be involved in the regulation of milk quality. GO analysis of their targets showed that functions associated with cellular process, transport, cellular biosynthesis, and gene expression were enriched in these miRNAs. Moreover, pathway analysis revealed that most target genes were related to signal transduction and lipid and amino acid metabolism. Persistent lactation is dependent on maintaining the number and activity of milk-secreting cells with advancing lactation^45^. Notably, several of the DE miRNAs (miR-100, miR-214, miR-182, miR-367b, miR-382, miR-330, miR-92b, miR-126-5p, miR-202, miR-369-5p and miR-31) have previously been reported to be involved in cell biology or metabolism^46–56^. Target prediction indicated that the phosphatase and tensin homolog (*PTEN*) gene is a putative target of miR-214,which has been reported to be involved in the regulation of cell survival and proliferation through modulation of *PTEN*^57,58^. Moreover, *PTEN* can down-regulate DCMEC-mediated secretion of beta-casein, triglyceride, and lactose and plays a critical role in lactation-related signaling pathways^59^. Furthermore, *PTEN* is a member of the PTEN-Akt pathway, which is required for lactation initiation and which provides a direct link between the *Akt* and *Stat5* pathways^60^. It has been reported that miR-214 is directly involved in lactoferrin expression in mammary epithelial cells^47^. However, miR-369-5p impairs adipogenesis by down-regulating *DNMT3A*^55^.

Moreover, to reveal the functions of the DE miRNAs, we performed an integrative analysis of miRNA and mRNA expression that provided a transcriptomic perspective of potential miRNA-mRNA interactions in the bovine mammary gland. It was demonstrated that 36 miRNAs had a negative regulatory relationship with 253 target mRNAs. Functional analysis indicated that these targets were involved in regulating milk quality. For instance, eleven members of the solute carrier (*SLC*) family participate in transport of amino acids, carbohydrates, glucose, dicarboxylic acid, ions, phosphate, phospholipids, urea, and water^61^. It is well known that amino acids are the raw materials of protein synthesis. Thus, the transport of amino acids, particularly essential amino acids^62^, is critical for the synthesis of milk proteins^61^. Moreover, FA desaturase 1 (*FADS1*) causes desaturation of biosynthesized FAs^63^. In addition, *LALBA*, *MFGE8*, *GPAM*, *RHOF* and *TMEM120B* were identified by RIP and qPCR. Recent studies reported that MFGE8, a glycoprotein that is a constituent of the milk fat globule membrane (*MFGM*)^64^, promotes the cellular uptake of FAs from serum in multiple organ systems^65^. GPAM, a key enzyme in the lipid biosynthesis of triacylglycerols and phospholipids, has been associated with changes in cellular metabolism and with increased synthesis of phospholipids in human breast cancer^66^. Based on previous predictions and the above analysis, miR-214 negatively regulates multiple genes, including *GPAM*, *MFGE8*, *LALBA*, *SLC20A2*, *SLC2A1* and *SLC7A5*, suggesting a role of miR-214 in milk quality. In summary, we investigated the DE miRNAs related to milk quality, and our evidence suggests that these miRNAs play roles in milk protein/fat biosynthesis. However, the mechanisms by which most of these DE miRNAs regulate milk quality remain unclear and need further verification.

## Conclusion

In this study, a comprehensive analysis of RIP-seq, small RNA-seq and mRNA-seq was performed. 38 DE miRNAs have been described as regulators of milk protein and milk fat, and 25 core DE miRNAs were implicated in epithelial cell development, mammary gland development, regulation of cell proliferation, amino acid transport, and lipid metabolism. We found many DE miRNAs have strong and novel associations with lactation. Finally, this rich dataset should serve as a valuable reference for further exploration of lactation.

## Materials and Methods

### Ethics statement

The animal protocols have been approved by the Animal Care and Use Committee, Northeast Agricultural University, Harbin, China, and all procedures were conducted in accordance with the approved protocol.

### Animal study and sample collection

All experiments were performed using 15 healthy multiparous Holstein cows with a similar genetic background. Of these cows, 12 were in the lactating period and 3 were in the dry period (D). All 12 lactating cows were in the third parity, with calving at 52 to 54 months of age and at 90 days in milk (DIM). Lactating cows were milked at 8 a.m. and 3:30 p.m. daily. The dry cows were pregnant and dried off at 310 DIM. All cows were fed a ration based on grass silage and concentrate (Table S1). The feeding regimen was ad libitum during lactation and restricted during the dry period in accordance with requirements. All animals had free access to fresh water. Lactating cows were allocated to the H group (milk protein ≥ 3% and milk fat ≥ 3.5%, n = 6) or the L group (milk protein ≤ 3% and milk fat ≤ 3.5%, n = 6) according to their milk protein and fat contents (Table 3). The results are shown as the means ± standard deviation (SD). Statistical comparisons were performed with unpaired two-tailed T-tests. Differences were considered significant for an adjusted *P* value ≤ 0.05.

**Table 3:** Cow milk compositions.

Milk component	High-protein/high-fat group	Low-protein/low-fat group
Milk protein (%)	3.28 ± 0.03	2.86 ± 0.02
Milk fat (%)	4.16 ± 0.02	3.37 ± 0.01
Lactose (%)	4.83 ± 0.03	4.53 ± 0.03
Dry matter (%)	11.91 ± 0.02	10.90 ± 0.04
Milk yield (kg/d)	33.91 ± 2.11	33.73 ± 0.52
Somatic cell (cells/ml)	≤ 50, 000	≤ 50,000

The results are shown as the means ± SD. Statistical comparisons were performed with unpaired two-tailed T-tests. Differences were considered significant if the adjusted *P* value ≤ 0.05.

Samples from the three groups were collected following the methods described by Hou *et al*.^67^. Briefly, these lactating cows were slaughtered at 90 DIM, and dry cows were slaughtered at 30 d after dry off. The lactating cows were milked 1 h before slaughter. Immediately after exsanguination, several pieces of mammary parenchyma tissue were aseptically removed from the midregion of the mammary glands. Tissue samples were trimmed of visible connective and adipose tissues. Small pieces of mammary tissue were frozen and immediately stored in liquid nitrogen at −80 °C until use. For mammary gland epithelial cell culture, fresh tissue was placed in ice-cold Hanks balanced salt solution (Life Technologies, USA) and then transported to the laboratory.

### Construction of RNA sequencing libraries and sequencing

Total RNA was extracted from ~100 mg of mammary gland tissues using TRIzol reagent (Invitrogen, USA) and was then cleaned up using the Qiagen RNeasy® Plus Mini Kit (Qiagen, USA) according to the manufacturer’s protocol. RNA quantity was assessed using a Nano-Drop ND-2000 UV-Vis Spectrophotometer (Thermo Fisher Scientific, Inc.), and an Agilent 2100 Bioanalyzer (Agilent Technologies) was used to assess the quality of RNA. RNA samples with an RNA integrity number greater than 7.5 were used. A TruSeq^®^ Small RNA Library Prep Kit (Illumina, USA) was used to prepare small RNA-seq libraries according to the manufacturer’s protocol. The Agilent 2100 Bioanalyzer and an Agilent High Sensitivity DNA chip (Agilent Technologies) were used to assess the miRNA-seq libraries. The 50-nt sequence tags were obtained via Illumina HiSeq. 2000 sequencing by the Beijing Genomics Institute (BGI, Shenzhen, China). TruSeq^®^ Stranded mRNA Sample Preparation Kits (Illumina, USA) were used to construct mRNA sequencing libraries according to the manufacturer’s protocol. The quality of the libraries was assessed using an Agilent 2100 Bioanalyzer and a DNA-specific chip such as the Agilent DNA 1000 (Agilent Technologies). The final product was expected to be a band at approximately 260 bp. High-throughput sequencing was performed by Novel Bioinformatics Co., Ltd. (Shanghai, China) using an Illumina NextSeq. 500 system.

### RIP and RIP-seq library preparation

RIP was performed using a Magna RIP™ RNA-Binding Protein Immunoprecipitation Kit (Merck Millipore, USA) according to the manufacturer’ s protocol. Purified RNA was measured using a Nano-Drop ND-2000 UV-Vis Spectrophotometer, and the Agilent 2100 Bioanalyzer was used to assess the quality of RNA. Following preparation of the reference RIP-seq and mRNA-seq libraries, RIP sequencing was performed by Personal Biotechnology Co., Ltd. (Shanghai, China) using an Illumina NextSeq. 500 system.

### Small RNA-seq data analysis

The reads were trimmed with Cutadapt v 1.9.1^68^. Reads shorter than 18 nucleotides were discarded. Quality control of the reads was performed using FastQC v 0.11.5. The ends of the filtered reads were then further trimmed to remove low-quality bases (Phred score ≤ 20). The clean reads were mapped to the bovine genome (Btau_4.6.1) using BWA v 0.7.13^69^. Furthermore, HTSeq v 0.6.1p2^70^ was used to count the number of uniquely aligned reads using Rfam and miRBase21. Differential expression analysis of miRNA expression profiles was quartile-normalized using EBSeq v 1.12.0^71^. miRNAs with an abundance of greater than one CPM in at least three of the samples were defined as true known miRNAs. miRNAs uniquely expressed in one tissue were defined as period-specific miRNAs. miRNAs expressed in all three groups were defined as commonly expressed miRNAs. miRNAs whose expression was significantly lower or higher in one group than in any other group (FDR ≤ 0.05^72^, |log_2_ (fold change)| ≥ 1) were referred to as DE miRNAs. We used the miRDeep2 package (Ver.2.0.0.5), which discovers miRNA genes by analyzing sequenced RNAs, to predict novel and known miRNAs. This tool reports known and novel microRNAs with high accuracy in seven species representing the major animal clades^73^.

### RIP-seq and mRNA-seq data analysis

FastQC was used to check the quality of raw reads. Cutadapt was used to remove the sequence joints of reads, and the FASTQ Quality Filter command was used to identify low-quality reads with a Phred score ≤ 20. Reads shorter than 50 bp in length were discarded. Finally, clean reads were mapped to the bovine genome UMD 3.1^74^ using Tophat2^75^ and Bowtie v 2.2.9 (https://sourceforge.net/projects/bowtie-bio/files/bowtie2/). HTSeq v 0.6.1p2 was used to count reads uniquely aligned to the Ensembl (version 84) annotation of the bovine genome. Differential expression analysis was performed with DESeq. 2^76^. The mapped read count was normalized according to the library size. Genes with |log_2_ fold change in expression| ≥ 1 and P-value ≤ 0.05 were defined as DEGs.

For functional analysis of DEGs, the DEGs were classified according to GO terms using the PANTHER Classification System^11^ and WEGO^77^, and they were annotated into KEGG pathways using the CapitalBio Molecule Annotation System (MAS 3.0, http://bioinfo.capitalbio.com/mas3/).

### Prediction and functional analysis of miRNA targets

The target genes of selected miRNAs were predicted using TargetScan Release 7.1^78^ or miRanda^79^. The target genes that were predicted by both miRanda (Total score ≥140, Total energy ≤− 15) and TargetScan (default parameters)^80^ for the selected miRNAs were further analyzed. GO^81^ analysis using PANTHER was conducted to understand the miRNA target genes and the potential roles of DEGs. The KEGG database was used to identify significant pathways related to the target genes^82^. The FDR was calculated to correct the P-value, and the FDR was then used to determine the significance of the predicted function. Thresholds of FDR ≤ 0.05 and enriched gene number ≥ 2 were applied to identify biological functions significantly enriched in the selected miRNAs. In addition, the correlation between miRNAs and mRNAs was analyzed, and if the correlation analysis showed a negative correlation, the gene was identified as a putative target for the candidate miRNA. Networks of miRNA-mRNA pairs were constructed with Cytoscape to better understand the relationships between novel miRNAs and mRNAs^83^.

### Network analysis and construction

Gene-Act network analysis was performed to identify the interactive network among the GO terms enriched in miRNAs negatively regulated by mRNAs based on the GO database using the BiNGO tool of Cytoscape, which calculates overrepresented GO terms in the network and displays them as a network of significant GO terms^84^. Gene pathway network analysis was performed to identify the relative enrichment of each pathway involved in signal transduction, cell cycle and metabolism based on the significance of the input DEGs according to the KEGG database. Gene correlation analysis was conducted based on the pathways from the KEGG database, which enabled the identification of two different genes in the same pathway of the same database. The STRING database was employed to analyze protein-protein interactions and to construct a Gene-Act network of the negative regulatory interactions between DE mRNAs and miRNAs based on the relationships extracted from the database, including activation, inhibition, binding, catalysis, phenotype, posttranslational modification, reaction, and transcriptional regulation^85^.

### qPCR

Total RNA from mammary gland tissues of 15 cows was synthesized into cDNA using the NCode™ miRNA First-Strand cDNA Synthesis Kit (Invitrogen, USA), and miRNA expression was measured using a Hairpin-it^TM^ miRNA qPCR Quantitation Kit (GenePharma, Suzhou, China) according to the manufacturer’s protocol. Alternatively, total RNA was synthesized into cDNA using a cDNA Synthesis kit (Exiqon, USA) to measure mRNA expression using SYBR® Green Master Mix (Exiqon, USA) according to the manufacturer’s protocol. The calculation of relative expression levels of selected miRNAs or mRNAs was conducted using the ΔΔCt method^86^, with U6 or β-actin as an internal control. The experiment was performed in triplicate. Statistical comparisons were performed with unpaired two-tailed T-tests. Differences were considered significant for an adjusted *P* value ≤ 0.05.

## Electronic supplementary material

Table 3

Supplementary Information
