# Supplementary material for: Comparative transcriptome analysis to investigate the potential role of miRNAs in milk protein/fat quality
Source: Sci Rep. 2018 Apr 19;8:6250. doi: 10.1038/s41598-018-24727-y (PMC5908868; doi:10.1038/s41598-018-24727-y)

Supplementary information

Comparative transcriptome analysis to investigate the potential role of miRNAs in milk protein/fat quality

Xuehui Wang, Li Zhang, Jing Jin, Anting Xia, Chunmei Wang*, Yingjun Cui, Bo Qu, Qingzhang Li and Chunyan Sheng

Key Laboratory of Dairy Science of Education Ministry, Northeast Agricultural University, Harbin 150030, Heilongjiang, China;

* Corresponding author: Chunmei Wang Key Laboratory of Dairy Science of Education Ministry, Northeast Agricultural University, Harbin 150030, Heilongjiang, China, Tel: +86 13936507139, E-mail: wangcm-1@163.com (W.C.)

Table S1. Concentrate composition of lactating and dry cow rations

**Concentrate composition (%)**	**Lactating**	**Dry**
Corn	/	67.5
Cottonseed	15	8
Rapeseed	8	6
Soybean meal	12	/
Extruded soybean	30	4
Wheat bran	/	10
Limestone	2	1.5
Sodium bicarbonate	2	1
Calcium bicarbonate	2.7	1
Sodium chloride	1.5	/
DDGS	24	/
Methionine	0.8	/
Mineral and vitamin premix	2	1

Figure S1. Functional enrichment analysis and pathways classification targets of differentially expressed miRNAs. (**A**) Functional enrichment analysis of targets by 38 differentially expressed miRNAs in H and L group. (**B**) Pathway classification of targets by 38 differentially expressed miRNAs in H and L group.

A

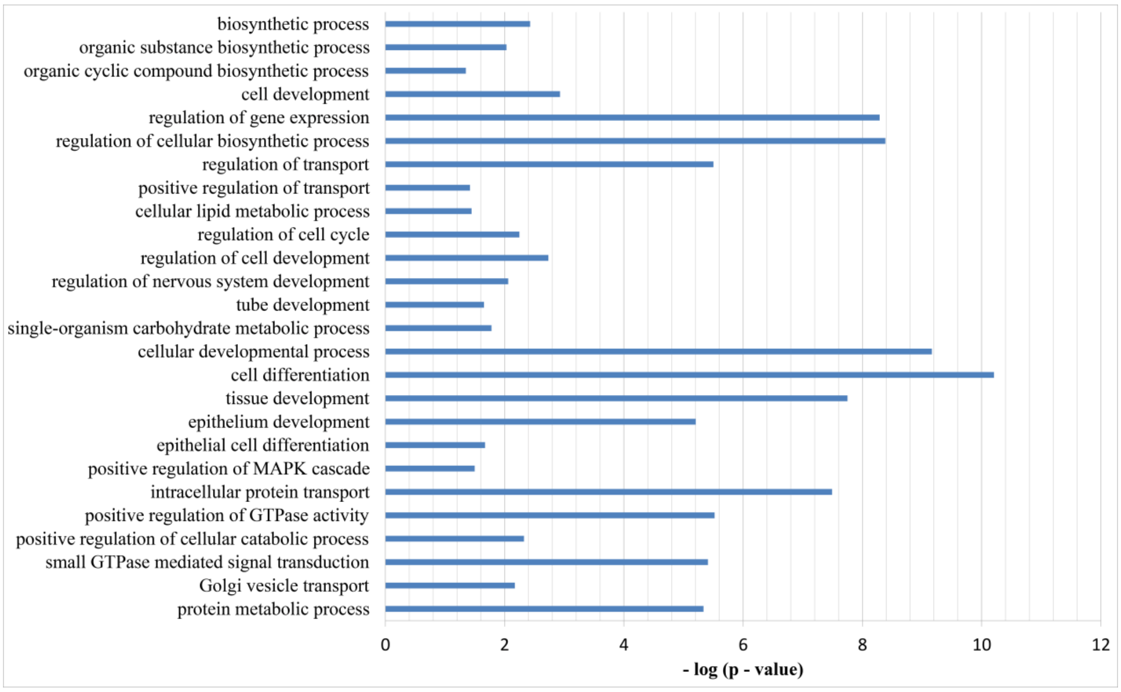

B

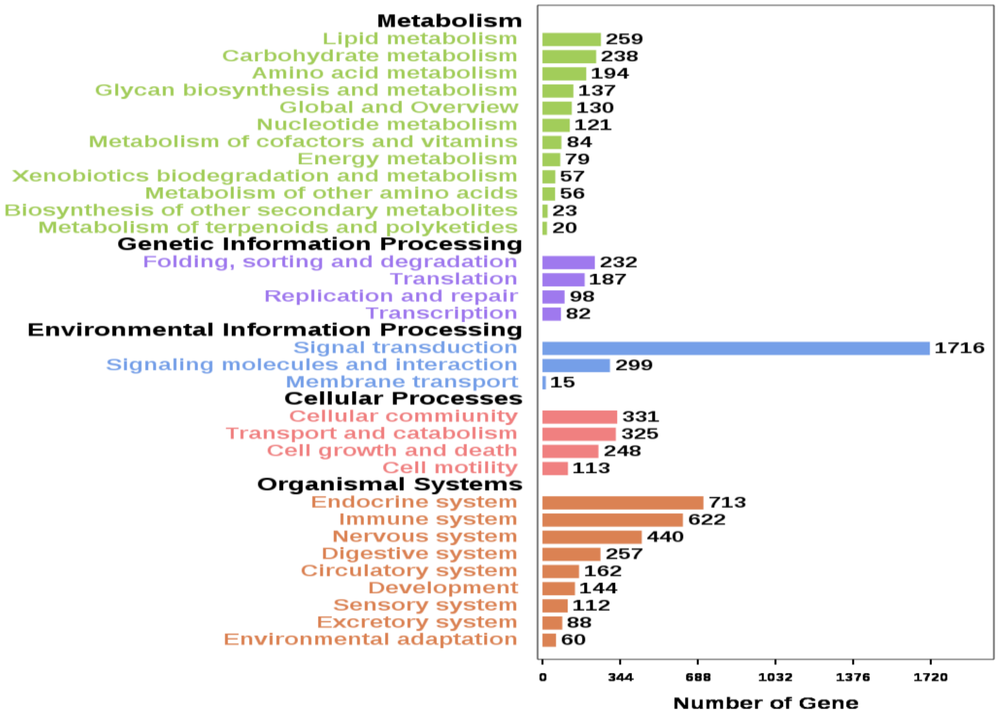

Figure S2. (**A**) Gene pathway network analysis results indicated that signal transduction, cell cycle and metabolism were the most important pathway in H vs. L of putative gene. (**B**) Gene correlation was analysis based on pathway from KEGG database, showing that most genes have one association on the same pathway of the same database.

A

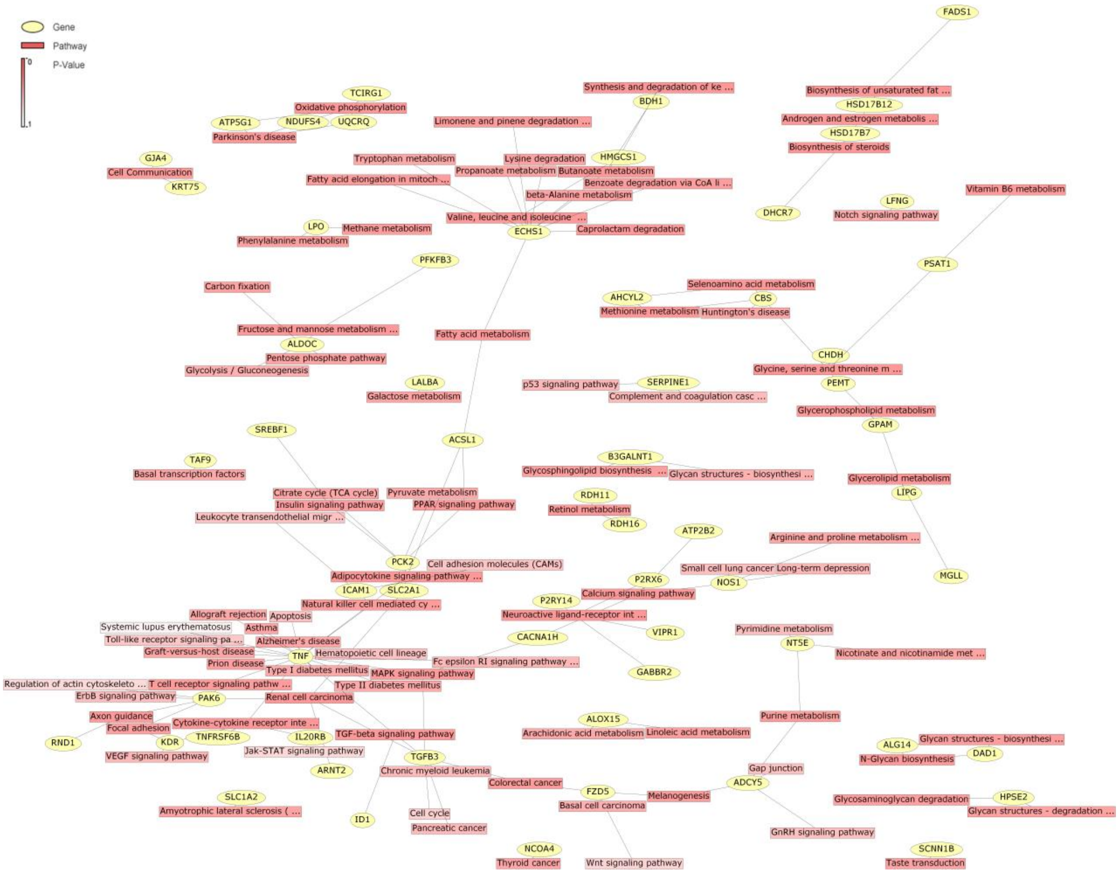

B

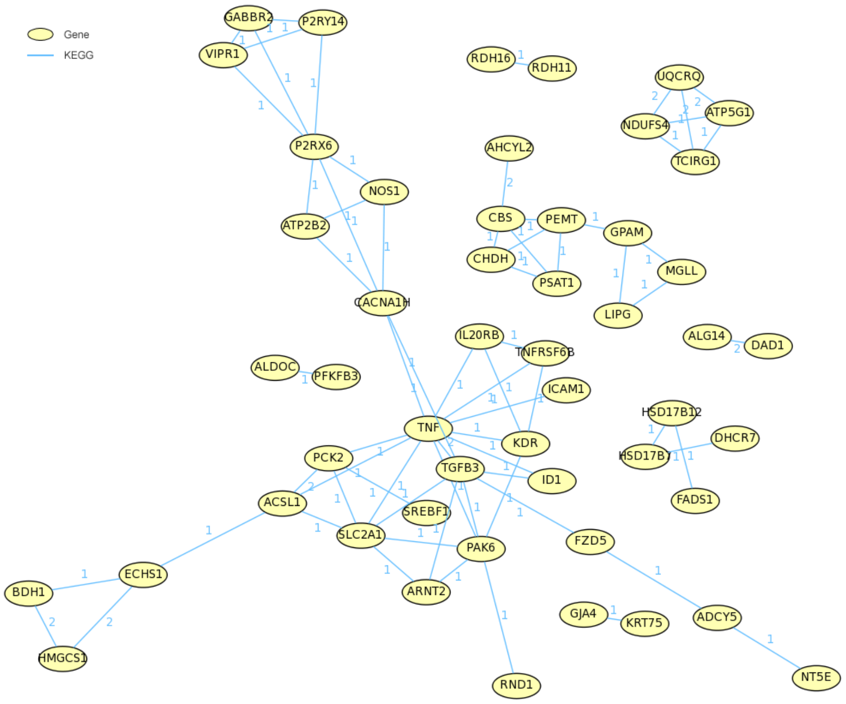

Table S2. RIP gene differentially expressed in L compared to H sample.

Gene symbol	Difference in log2 FC (L / H)	P value
LALBA	2.82	4.99E-07
ABCG2	2.51	7.52E-06
HSTN	2.47	7.78E-05
CSN1S1	1.98	2.77E-04
TMEM120B	1.69	1.83E-03
WNT5A	-2.24	2.22E-03
FASN	1.56	3.70E-03
PAEP	1.48	5.69E-03
CSN1S2	1.45	6.93E-03
SLC34A2	1.44	7.31E-03
RHOF	1.45	7.66E-03
SLC39A8	1.37	1.15E-02
CSTB	1.53	1.50E-02
MFGE8	1.26	1.82E-02
GPAM	1.19	2.59E-02

Figure S3. qPCR validation of the miRNAs and mRNAs expression. (**a**) L compared to D, (**b**) H compared to D, (**c**) H compared to L, (**d**) L compared to H of RIP – seq and mRNA – seq, L compared to H of qPCR.

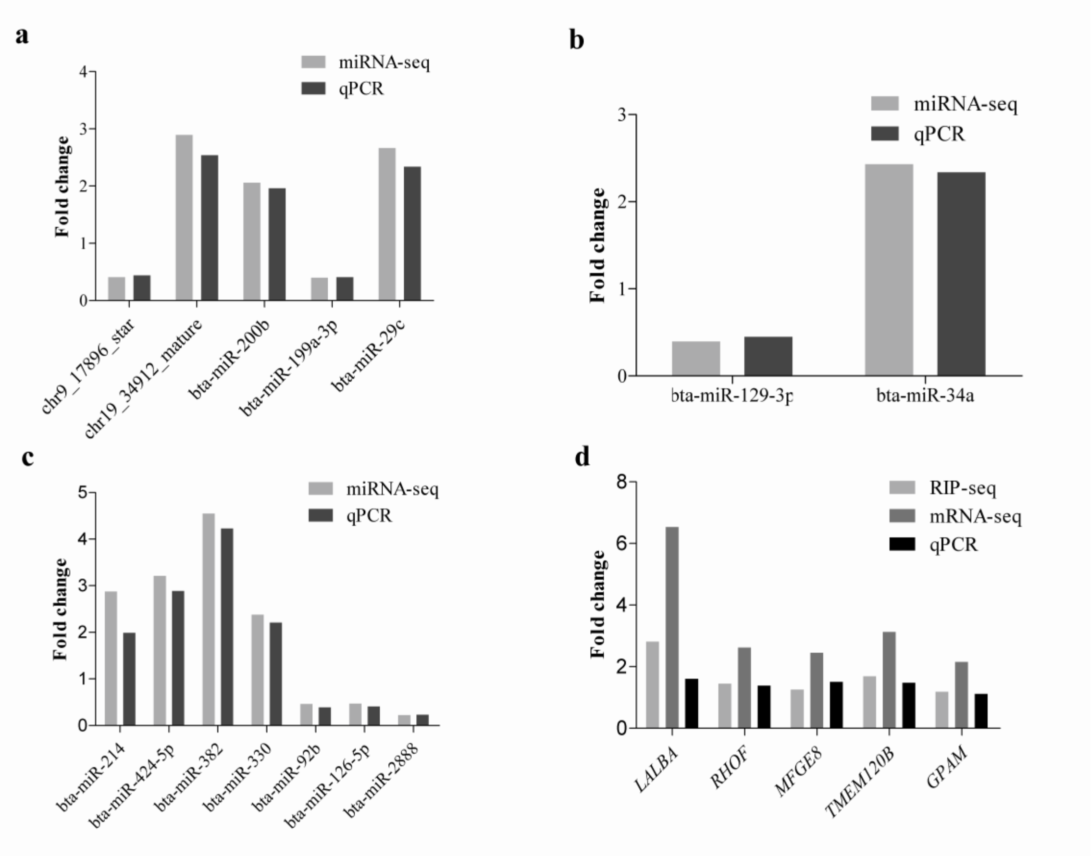

Supplement: Supplementary file 2 — Supplementary Information [file 41598_2018_24727_MOESM2_ESM.pdf]
